# Imaging of the knee in juvenile idiopathic arthritis

**DOI:** 10.1007/s00247-017-4015-6

**Published:** 2018-05-08

**Authors:** Robert Hemke, Nikolay Tzaribachev, Anouk M. Barendregt, J. Merlijn van den Berg, Andrea S. Doria, Mario Maas

**Affiliations:** 1 Department of Radiology and Nuclear Medicine, Academic Medical Center, University of Amsterdam, Meibergdreef 9, 1105AZ Amsterdam, The Netherlands; 2 Pediatric Rheumatology Research Institute, Bad Bramstedt, Germany; 3 Department of Pediatric Hematology, Immunology,Rheumatology and Infectious Disease,Emma Children’s Hospital AMC, University of Amsterdam, Amsterdam, The Netherlands; 4 Department of Diagnostic Imaging, The Hospital for Sick Children, University of Toronto, Toronto, ON Canada

**Keywords:** Children, Juvenile idiopathic arthritis, Knee, Magnetic resonance imaging, Radiography, Ultrasound

## Abstract

In juvenile idiopathic arthritis (JIA), imaging is increasingly used in clinical practice. In this paper we discuss imaging of the knee, the clinically most commonly affected joint in JIA. In the last decade, a number of important steps have been made in the development of imaging outcome measures in children with JIA knee involvement. Ultrasound is undergoing a fast validation process, which should be accomplished within the next few years. The validation processes of MRI as an imaging biomarker for clinical trials in the JIA knee are at an advanced stage, with important data available on the feasibility, reliability and validity of the Juvenile Arthritis MRI Scoring system. Moreover, both US and MRI data are emerging on the normal appearance of the growing knee joint.

## Introduction

The knee joint is the most commonly affected joint in juvenile idiopathic arthritis (JIA) [1]. It can, therefore, be considered an index joint for evaluation of disease and for monitoring response to therapy. As in other joints, knee involvement in the scope of JIA is characterized by a swollen, painful, warm joint with loss of function.

Conventional radiography has played an important role in the management of JIA, but imaging techniques such as ultrasonography (US) and MRI are now considered more helpful for several reasons. First, the trend towards early suppression of inflammation to prevent irreversible damage of cartilage and bone has shifted the emphasis from detecting damage (using radiography) to detecting early joint changes of JIA. This drives the need for imaging techniques that are more sensitive than radiography in the evaluation of inflammatory processes as well as early osteochondral changes. In this regard, MRI and US play an increasingly important role in evaluating and monitoring disease activity [2]. Second, the physical examination remains the reference standard for identifying disease activity in both daily practice and in clinical trials. However, physical examination has limited reliability, even if performed by an experienced observer [3], underpinning the potential of imaging in helping clinical decision-making. Moreover, advances in therapies have increased the number of children who reach clinically inactive disease. Further, knowledge about subclinical inflammation and its influence on the child’s outcome is rising [2].

## Conventional radiography

In the clinical setting, conventional radiography still plays an important role, especially in narrowing the differential diagnosis and in establishing a baseline for disease follow-up. Although radiography provides important information on growth disturbances and damage to cartilage and bone, it does not show early changes suggestive of active inflammation [4]. No validated scoring methods are available for evaluating JIA knee disease activity using radiography. There is little information on reliability and validity of scores for use in children or on the potential limitations of radiographic scoring systems for assessing growing joints because of the measurement properties of the scales [5].

Albeit nonspecific, the presence of joint fluid and synovial thickening can be seen as increased density in the infrapatellar fat pad and suprapatellar region. In the Western population, bone erosions in knee joints in children with JIA are relatively rare. Because of the availability of more effective treatment options and the relatively large amount of epiphyseal cartilage in knees in growing children, erosive damage is usually only seen as a late complication of JIA. When present, bone cysts and bone erosions can be seen. Moreover, loss of articular cartilage can cause gradual joint space narrowing. Joint malalignment and ankyloses in the knee are extremely rare.

Radiography does have an advantage over ultrasound and MRI in the determination of growth disturbances. Due to hyperaemia, overgrowth of extremities and disturbance of epiphyseal bone formation can be observed. Moreover, it is easier to compare bilateral joints using radiography compared to, for instance, MRI.

## Ultrasound

In paediatric rheumatology, ultrasound plays an important role in narrowing the differential diagnosis and can be useful for treatment monitoring as well as for guidance for joint injections [6]. It is superior to clinical examination in diagnosing disease activity and in detecting subclinical disease [7]. Because of its relatively low cost and wide accessibility, it allows for assessment of multiple joints within scanning times that vary according to the level of detail required for the examination. Limitations to the method include its inability to examine bone marrow or to reliably detect central erosive changes given the low penetration of the ultrasound beam to the central aspect of the joint with high-frequency transducers [8].

### Acquisition techniques and ultrasound definitions

For the application of US in paediatric rheumatology, both grey-scale B-mode and power/colour Doppler modes should be used in every examination. Also, standard scanning positions should be considered. For standard clinical paediatric ultrasound examination of the knee, the child is placed in supine position with a slightly flexed knee. The standard sagittal view includes the patella inferiorly, the quadriceps tendon, and the suprapatellar recess [9]. An axial view in the popliteal fossa can be considered to exclude a Baker cyst [9]. In the scope of research a more extensive ultrasound protocol of the knee can be considered (Table 1).

**Table 1:** Knee ultrasound technique

	Plane 1 (anterior suprapatellar recess)*	Plane 2 *(lateral parapatellar recess)*	Plane 3 *(infrapatellar region)*	Plane 4 *(condylar cartilage)*
Positioning knee joint	30° flexion	Knee straightened, patella in a central position	30° flexion	90° flexion
Positioning transducer	Longitudinal to the suprapatellar recess/the quadriceps tendon	90° transversal to plane 1	Longitudinal to the infrapatellar tendon and the tibial tuberosity	Transversal to plane 1
Anatomical landmarks	Superior patellar edge, suprapatellar fat pad, quadriceps tendon, femur and pre-femoral fat pad	Superior patellar edge, femoral condyles, suprapatellar fat pad	Inferior patellar edge, infrapatellar tendon, Hoffa fat pad, tibial tuberosity	Quadriceps tendon, suprapatellar fat pad, condylar cartilage
Features to assess	Synovial effusion, synovial hypertrophy, synovial vascularity (hyperperfusion), erosive changes, morphology and structure of the tendons, vascularity of the tendon and the tendon sheet	Synovial effusion, synovial hypertrophy, synovial vascularity (hyperperfusion), erosive changes	Synovial effusion, morphology and structure of the Hoffa fat pad, vascularity of the Hoffa fat pad, morphology and structure of the tendon, vascularity of the tendon, morphology and structure of the tibial tuberosity, vascularity of the tibial tuberosity	Morphology and structure of the cartilage, erosive changes

*Can alone be considered as a standard clinical paediatric ultrasound plane

**Fig. 1:**
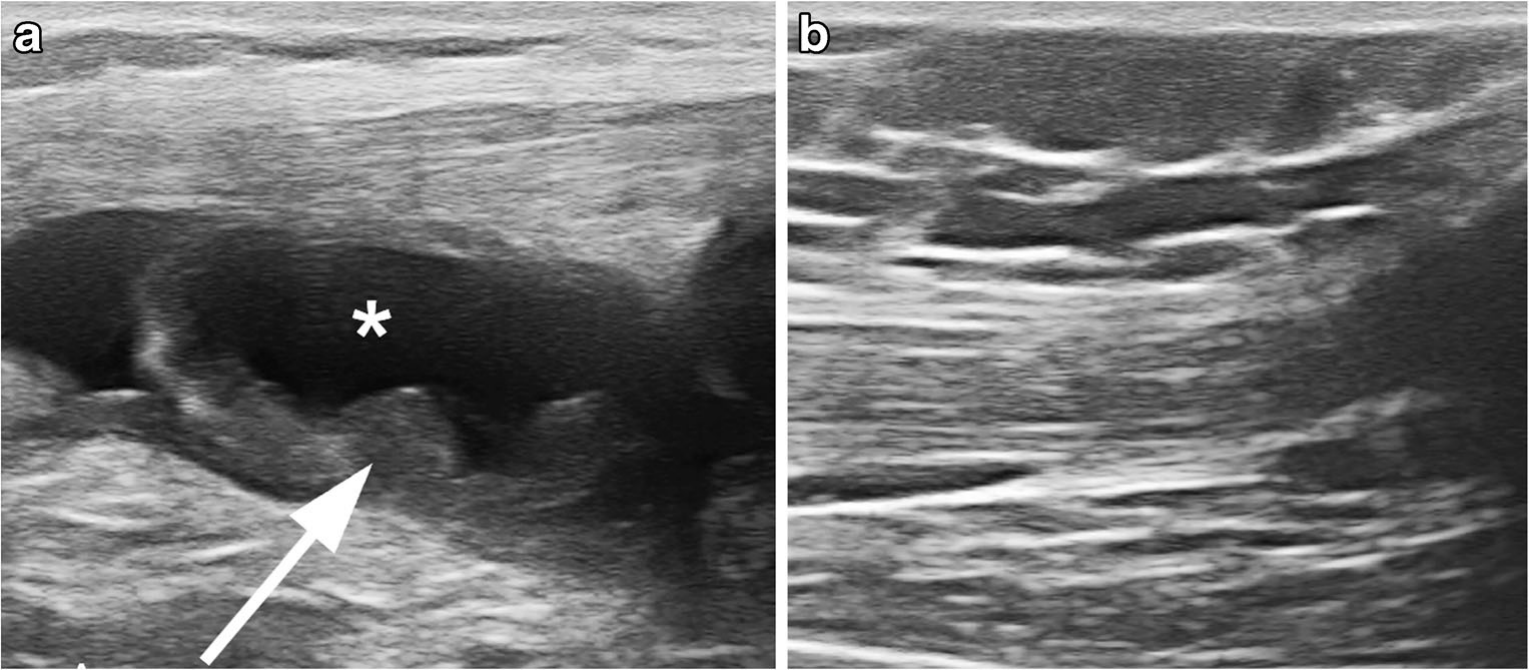
Ultrasound longitudinal to the suprapatellar recess in an 8-year-old girl with juvenile idiopathic arthritis. **a** There is evident synovial hypertrophy (*arrow*) and joint fluid in the suprapatellar recess (*). **b** After several months of treatment, the synovial hypertrophy and joint fluid have disappeared

The main US features characterizing pathology in JIA at this stage of the development of a US-based outcome measure are *synovial thickening* and *synovial effusion* [10]. Synovial thickening (Fig. 1) is defined as abnormal, intra-articular, hypoechoic material that is non-displaceable. Synovial effusion (Fig. 1) is defined as an abnormal, intra-articular, anechoic or hypoechoic material that is displaceable. When looking for a synovial effusion, one should be aware that a change of patient position can influence the level of effusion detected. Moreover, a scanning technique avoiding excessive pressure is warranted because joint fluid can shift to another synovial recess when too much pressure is used [11].

Power/colour Doppler is useful to acquire information about tissue perfusion. It is important to clarify that synovitis can be detected on the basis of B-mode findings (synovial thickening or synovial effusion) alone. Nevertheless power and colour Doppler have proved to be useful in differentiating active (hypervascular) from fibrotic (hypovascular) pannus [11]. In growing children, the sole detection of mildly increased vascularity does not allow for the diagnosis of synovitis because of the different levels of joint vascularity according to a child’s age and level of physical activity of the joint [12]. It is important to recognize the presence of such normal vessels in paediatric joints from normal maturation and these should not be considered pathological [13].

In children with JIA, *loss of cartilage thickness* has been described. US has a strong correlation with MRI in the evaluation of cartilage thickness of the medial and lateral condyle and the intercondylar region, with a rho correlation of 0.70–0.86 [14].

### Scoring system

We are currently developing a consensus-based US scoring system. The preliminary scoring system consists of three grades separated for synovitis and synovial perfusion (Table 2). Its reliability will be tested in a patient-based exercise.

**Table 2:** Suggested scoring system of ultrasound for the knee joint

Feature	Definition	Locations	Scale
Synovitis B mode	Comprises synovial hypertrophy and synovial effusion	Suprapatellar recess, parapatellar recess	Grade 1 = mild Grade 2 = moderate Grade 3 = severe
Synovitis power/colour Doppler mode	Comprises synovial hypertrophy and hypervascularity*	Suprapatellar recess, parapatellar recess	Grade 1 = mild Grade 2 = moderate Grade 3 = severe

*Note that hypervascularity without synovial hypertrophy does not count for synovitis

Synovitis = synovial hypertrophy and/or synovial effusion. Synovial hypertrophy is defined as abnormal, intra-articular, hypoechoic material that is non-displaceable. Synovial effusion is defined as abnormal, intra-articular, or hypoechoic material that is displaceable

### Normal values

Age-related US findings have been described in healthy children [13, 15]. There are considerable age-related variations in children’s joints, including the knee examined by US, where Doppler depicts vascularity, particularly within the epiphyseal cartilage of the children at a younger age. Also, age- and gender-related differences and standard reference values of the cartilage thickness of the knee in healthy children have been elaborated [16]. However, the number of studies focusing on normal ultrasound values of the knee in children is limited. Therefore the normal appearance of the synovial membrane, for instance, should be interpreted with care.

## Magnetic resonance imaging

Within the last decade, the use of MRI and advances in MRI techniques have substantially improved the evaluation of joint pathologies in children with JIA [4]. MRI is the preferred imaging modality for the assessment of inflammatory and destructive changes in JIA as compared to conventional radiography, ultrasonography and physical examination [3, 5]. Furthermore, MRI is the only imaging modality that is useful in visualizing bone marrow changes, a potential predictor of erosive joint damage in JIA, as previously demonstrated in adults [17]. Therefore despite practical limitations, MRI holds the potential to become an important outcome measure for assessing the knee joint in clinical trials of children with JIA.

### Main MRI features and imaging protocol

In children with JIA and knee involvement, the main imaging features include synovial thickening, joint effusion and bone marrow oedema. Moreover cartilage loss and bone erosions — although relatively rare in paediatric patients — can be observed. Tendinopathy, enthesopathy, inhomogeneity of the infra-patellar fat pad and bone cysts can be seen, as well, but these abnormalities are relatively uncommon in the knee joint. Moreover, the reliability of scoring these secondary features is unsatisfactory [18]. Table 3 shows an MRI protocol for evaluating the JIA knee joint.

**Table 3:** Knee MRI protocol

Sequence	Plane	Goal	Required or optional
T2 FS or STIR (mDixon)*	Sagittal	Joint effusion, bone marrow oedema, bone erosions	Required
T2 FS or STIR (mDixon)*	Coronal	Bone marrow oedema, bone erosions	Required
T1 (mDixon)*	Coronal	Bone marrow oedema, bone erosions	Required
Gradient echo / PD	Sagittal	Cartilage loss	Required
T1 FS post-Gd	Axial	Synovial thickening, joint effusion	Required
Gradient echo (3-D)	Axial	Cartilage loss	Optional
T1 FS pre-Gd	Axial	Synovial thickening, joint effusion	Optional
T1 FS post-Gd	Sagittal	Synovial thickening, joint effusion	Optional

*mDixon best option if available

*FS* fat-suppressed, *Gd* gadolinium, *PD* proton density, *STIR* short tau inversion recovery, *T1* T1-weighted spin echo, *T2* T2-weighted

**Fig. 2:**
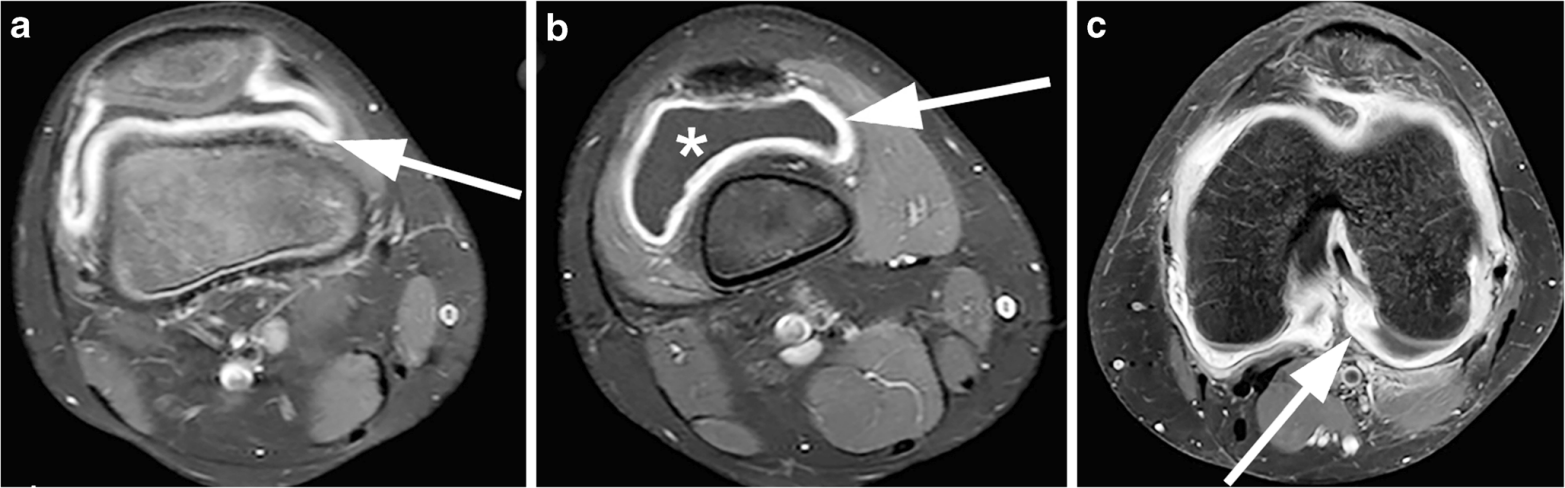
Axial T1-weighted MRI of the knee with fat suppression after gadolinium-based contrast administration in three patients with juvenile idiopathic arthritis shows synovial hypertrophy in the central locations of the knee. **a** Enhancing synovial hypertrophy in the retropatellar region (arrow) in an 9-year old girl. **b** Enhancing synovial hypertrophy in the suprapatellar region (arrow) in an 11-year old boy. Notice the non-enhancing low-signal-intensity joint fluid in thesuprapatellar recess (*). **c** Enhancing synovial hypertrophy around the cruciate ligaments (arrow) in a 15-year old girl

Synovial inflammation leading to *synovial thickening* is the principal pathological process in JIA, and the presence of synovial thickening on knee MRI is associated with the clinical onset of JIA [19]. On MRI, the inflamed synovial membrane is thickened and irregular, and its outline may be wavy. In the knee, a synovial thickness of >2 mm is considered pathological. The central locations in the knee (around the cruciate ligaments and retropatellar and suprapatellar areas, Fig. 2) are most commonly affected [20]. The signal intensity of this thickened synovial membrane is low to intermediate on T1-weighted images and high on T2-weighted images, similar to joint effusion. T1-weighted images after administration of gadolinium-based contrast agents provide better differentiation between joint effusion and synovial thickening [21]. Omitting intravenous gadolinium-based contrast agents in the MRI assessment of joints in JIA is therefore not advised.

Although not specific for JIA, *joint effusion* (Fig. 2) can frequently be found in children with JIA [22]. On MRI, effusion demonstrates high signal intensity on fluid-sensitive images and low signal intensity on T1-weighted images. Joint effusions are predominantly located in the suprapatellar and central joint recesses.

**Fig. 3:**
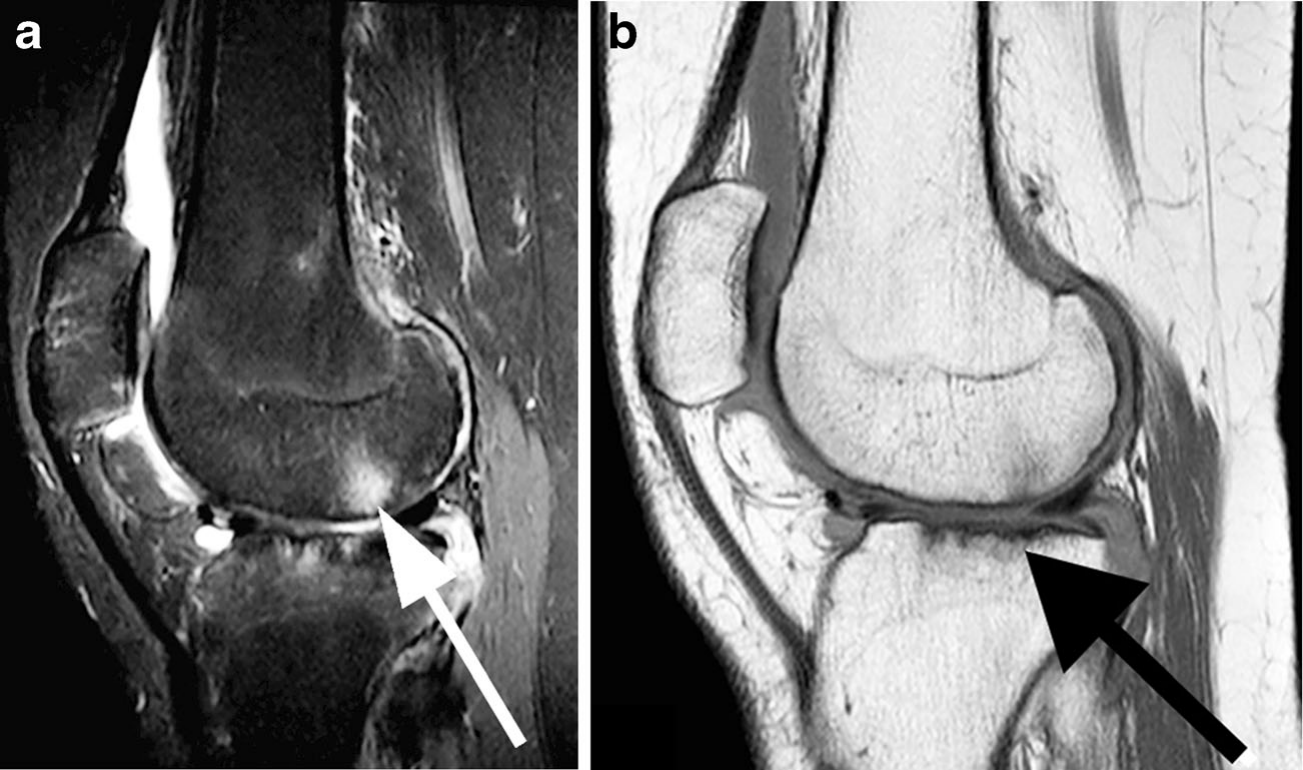
Sagittal MR images of the knee in a 16-year-old girl with poly-articular juvenile idiopathic arthritis. **a** T2-weighted sagittal MRI with fat suppression shows bone marrow oedema in the femur (*arrow*) and tibial plateau. **b** T1-weighted sagittal MRI shows bone erosions in the tibial plateau (*arrow*), with irregular signal intensity of the cortical bone and loss of the normal high signal intensity of trabecular bone

MRI is the state-of-the-art imaging technique to visualize changes in bone marrow suggestive of *bone marrow oedema* (Fig. 3). Bone marrow oedema can be seen as lesions with poorly defined margins within trabecular bone. It is marked by high signal intensity on T2-weighted fat-saturated images and low signal intensity on T1-weighted images. In rheumatoid arthritis, longitudinal studies have shown that the presence of bone marrow oedema is a key predictor of early erosive joint damage in adults with rheumatoid arthritis [17]. Therefore bone marrow oedema and synovial thickening are considered to be the most sensitive MRI features for monitoring disease activity in rheumatoid arthritis [23–25]. However to our knowledge there are a paucity of longitudinal studies, if any, focused on the prognostic value of bone marrow oedema in children with JIA.

*Cartilage loss* may be seen as areas of increased water content (proton-density/T2 hyperintense) in the articular cartilage or contour abnormalities, defects or thinning of the cartilage. Gradient-echo and proton-density-weighted sequences provide good contrast in the cartilage structure and can be used to evaluate the cartilage–synovial fluid interface and the subchondral bone.

MRI clearly shows the difference between changes in the articular cartilage and *bone erosions*. Bone erosions can be seen on T1-weighted images as loss of the normal low signal intensity of cortical bone and loss of the normal high signal intensity of trabecular bone (Fig. 3). Typically they have sharp margins. On T2-weighted images bone erosions appear as hypointense lesions, in contrast to subchondral cysts, which show as hyperintense signal on fluid-sensitive images.

### Scoring methods

Although MRI is the preferred imaging modality for detecting both inflammatory as well as destructive changes in JIA, international experiences with the use of MRI as an outcome measure in JIA are limited. Subsequently, this technique is underused in both clinical practice and research. In order to further internationally validate MRI as an outcome measure in JIA, a special interest group on Outcome Measures in Rheumatology on MRI was created in 2011 [26]. Within this special interest group on MRI in JIA, three working groups were initiated for further assessment of MRI scoring systems: one group focuses on temporomandibular joints, another on small joints and the third on large joints (primarily the knee joint).

### Reliability

In the last decade, two MRI scoring systems have been developed for the assessment of the paediatric knee joint: (1) the Juvenile Arthritis MRI Scoring system for use in JIA [27] and (2) the International Prophylaxis Study Group MRI Scoring system focusing on haemophilic arthropathy [28].

Recently the Outcome Measures in Rheumatology (OMERACT) special interest group on MRI in JIA conducted a reliability study evaluating both scoring methods [18]. This study showed good inter-observer reliability for MRI scores focusing on active disease (e.g., synovial thickening, joint effusion and bone marrow changes) and moderate to substantial inter-observer reliability for scores focusing on damage (e.g., cartilage lesions, bone erosions). Finally, the group proposed a combined juvenile arthritis MRI scoring system (JAMRIS) — shown in Table 4 — including items from both the original Juvenile Arthritis MRI Scoring and the International Prophylaxis Study Group systems [18].

**Table 4:** Combined juvenile arthritis MRI scoring system

Feature	Definition	Locations	Scale
Synovial thickening^a^	An area of the synovial compartment that shows a thickened synovial membrane and which can show enhancement after intravenous gadolinium administration	Six locations: patellofemoral area, suprapatellar recesses, infrapatellar fat pad, adjacent to the anterior and posterior cruciate ligaments, medial posterior condyle, and lateral posterior condyle	(0) normal, ≤2 mm (1) mild, >2 mm to ≤4 mm (2) moderate/severe, >4 mm Totals result in a minimum score of 0 and a maximum score of 12
Joint effusion^b^	An increased amount of fluid within the synovial compartment with high signal intensity on T2-weighted images and low signal intensity on T1-weighted images. Joint effusion has no post-gadolinium enhancement	The maximal diameter of the largest pocket of joint effusion is scored	(0) normal, ≤3 mm (1) mild, >3 mm to ≤5 mm (2) moderate/severe, >5 mm Totals result in a minimum score of 0 and a maximum score of 2
Bone marrow oedema^a^	An abnormality within the trabecular bone of the epiphysis, with ill-defined margins and high signal intensity on T2-weighted fat-saturated images and low signal intensity on T1-weighted images	Eight locations: lateral patella, medial patella, medial femur condyle, lateral femur condyle, medial weight-bearing region of the femur, lateral weight-bearing region of the femur, medial tibia plateau, lateral tibia plateau	Presence of bone marrow edema is scored semi-quantitatively based on the subjectively estimated percentage of involved bone volume at each site as follows: (0) none (1) <10% of the whole bone volume (2) ≥10–25% of the whole bone volume (3) >25% of the whole bone volume Totals result in a minimum score of 0 and a maximum score of 24
Cartilage loss^b^	Loss of cartilaginous tissue either focally (superficial or deep) or diffusely	Scored at the most severely affected location	(0) none (1) any loss (2) >50% volume loss (3) full-thickness loss (4) full-thickness loss >50% of surface Totals result in a minimum score of 0 and a maximum score of 4
Bone erosions^b^	A sharply marginated bone lesion with correct juxta-articular localization, typical signal characteristics and visible in two planes with a cortical break in at least one plane. On T1-weighted images there is a loss of the normal low signal intensity of cortical bone and loss of the normal high signal intensity of trabecular bone	Scored at the most severely affected location	(0) none (1) mild, any loss (2) moderate/severe, >50% surface involvement Totals result in a minimum score of 0 and a maximum score of 2

^a^Original Juvenile Arthritis MRI Scoring item

^b^Original International Prophylaxis Study Group item

Besides this international reliability study, the intra-observer and inter-observer reliability of the original Juvenile Arthritis MRI Scoring system has been evaluated, and both proved to be reliable [27]. These data are trustworthy, especially regarding MRI features focusing on active disease. Caution is needed with regard to MRI characteristics focusing on damage because these features are relatively uncommonly observed in JIA patients.

### Feasibility

Although MRI has some practical limitations such as limited availability in some regions/countries and the challenge of maintaining the same position for a prolonged period of time, it has proved feasible to perform contrast-enhanced knee MRI in children with JIA as young as 5 years old without the use of anaesthesia or sedation [29]. Moreover the use of the original Juvenile Arthritis MRI Scoring system proved feasible because the scoring takes an acceptable median of 6.6 min per patient [21].

### Construct and clinical validity

Within the last few years, two studies have focused on the sensitivity to changes in disease activity using the original Juvenile Arthritis MRI Scoring system [27, 30]. In both studies, with a follow-up period of 1 year, improvement of clinical JIA disease activity scores was associated with a significant decrease in MRI-based synovial thickening scores. These results represent good responsiveness of the Juvenile Arthritis MRI Scoring system, which is an important measure of validity. Moreover the Juvenile Arthritis MRI Scoring synovial thickening score proved useful in discriminating clinically active from clinically inactive JIA patients [31], indicating good discriminant validity.

Next to the responsiveness and discriminant validity, the clinical validity has also been evaluated. In a study focusing on whether clinical, laboratory or MRI measures were able to differentiate JIA with active arthritis from other causes of non-infectious arthritis in a group of patients with clinical signs of early arthritis, multivariate analysis showed that MRI-based synovial thickening was independently associated with JIA (odds ratio [OR] 6.58, 95% confidence interval 2.36–18.33) [19].

### Normal values

Because synovial inflammation is the hallmark of disease activity in JIA, it is important to determine the normal appearance and thickness of the *synovial membrane* in children. As stated, an intravenous injection of a gadolinium-based contrast agent is warranted for reliable evaluation of the synovial membrane. Therefore data on normal values for the synovial membrane in healthy children are sparse. Two studies that focused on the contrast-enhanced appearance of the synovial membrane in children have been performed: (1) A study by Nusman et al. [32] looked at the synovial membrane of the knee in children with inflammatory bowel disease who were clinically unaffected by arthritis; (2) Hemke et al. [33] determined the synovial membrane in knees of healthy children, using a standardized imaging protocol with post-contrast images obtained in the early phase (<5 min). The latter study showed that the normal synovial membrane thickness measures a maximum of 1.8 mm. The membrane was thickest around the cruciate ligaments, and retropatellar and suprapatellar recesses. The study by Nusman et al. showed a thickened synovial membrane (>2 mm) in more than half of patients with inflammatory bowel disease; however they did not observe any synovial thickness >4 mm. In all probability, the results from Hemke et al. are a more valid reflection of the true normal appearance of the synovial membrane because this study included healthy children (compared to patients with inflammatory bowel disease) and obtained post-contrast images in the early phase. Therefore, the Juvenile Arthritis MRI Scoring cut-off value of >2 mm for synovial thickening can be considered a valid measure.

Some *joint fluid* in the knee can be seen in the majority of healthy children. The largest pockets of normal joint fluid in healthy children are located in the central locations of the knee — around the cruciate ligaments and retropatellar region [33]. The mean diameter of the largest pocket of joint fluid in knees of healthy children is about 3 mm [15, 33].

*Bone marrow changes* suggestive of bone marrow oedema in knees of healthy children are relatively uncommon. In a study of 57 healthy children, bone marrow changes suggestive of bone marrow oedema were observed in 3 healthy children only. In all three children, the bone marrow changes were located in the apex patellae [33]. The presence of bone marrow changes suggestive of bone marrow oedema in the apex patellae in children with JIA should, therefore, be interpreted with care.

Moreover, zones of hematopoietic red bone marrow in the distal diaphysis and metaphysis of the femur might be seen as flame-like regions with signal characteristics fitting with bone marrow oedema. Typically, these marrow flames originate from the physis and have straight vertical margins [34]. These hematopoietic bone marrow flames are a normal finding in growing children and should not be mistaken for bone marrow oedema. So-called speckled bone marrow is another normal variant that might be mistaken for pathology. Small spots with bone marrow oedema signal characteristics are predominantly located in the feet and ankles of children younger than 15 years, although the spots can be seen in the tibia plateau and the distal epiphysis of the femur as well [35]. The speckled appearance might be caused by focal regions of residual hematopoietic bone marrow or physiological stress, possibly related to weight-bearing or altered biomechanics during normal growth.

The thickness of the normal articular cartilage in knees of growing children differs with age. As expected, the cartilage is normally thicker in younger children compared to older children. A study performed by Keshava et al. [15] clearly demonstrates these differences among different age groups in healthy boys. Moreover they showed that the normal thickness of the articular cartilage differs per location within the knee joint [15]. In the distal femur, ossification usually starts in the centre of the cartilaginous epiphysis. Because the newly formed ossification centre contains hematopoietic bone marrow, its signal intensity is the same as that of red marrow in the adjacent distal femoral metaphysis [36]. The ossification centre enlarges from endochondral bone development. Adjacent cartilage cells undergo hypertrophy during endochondral ossification, which results in increased signal intensity on T2-weighted images [34]. This area of normal high signal intensity is most obvious in the posterior part of the distal femoral epiphysis and can be quite discrete [37]. Furthermore, it is important to be aware of the high prevalence of ossification variants of the femoral condyle among boys ages 2–12 years and girls ages 2–10 years; this should not be considered pathological [37].

## Future work

The validation process of US in children with JIA is moving forward. Despite the relatively easy applicability of US, until now there has been no specific US knee scoring system. Rather the US knee examination is part of a more global US score covering multiple joints with respect to disease activity. The Outcome Measures in Rheumatology paediatric ultrasound working sub-group has performed a number of steps towards the validation of US as an outcome measure in paediatric rheumatology. Next important steps of the work of the sub-group include multicentre reliability testing of the newly created score tested in a single-centre prospective study. In this case, sensitivity to change will also be tested. The sub-group has started its work in tendons and entheses and should follow the Outcome Measures in Rheumatology Filter 2.0 criteria for the validation process. Another important step will be to develop scores incorporating osteochondral changes, where US is expected to be beneficial in evaluating peripheral cartilage. Its utility in detecting erosive changes might also be tested, and compared to that of MRI and radiography. This underlines the importance of close collaboration between US and MRI working groups, which is planned to start in 2018 with a joint symposium.

Although the first steps towards developing evidence-based guidelines for MRI data acquisition and interpretation have been made, further cooperation is necessary. The use of a juvenile arthritis MRI scoring system for the knee as an outcome measure in daily practice and clinical trials is promising. Thus far, juvenile arthritis MRI scoring has only been internationally tested in JIA patients visiting academic paediatric rheumatology centres in the Netherlands and Canada with full access to high-quality treatment. This has resulted in a population of studied JIA patients with only mild to moderate disease activity. Consequently, the presence of destructive changes of cartilage and bone has been relatively low in the studies performed until now. To evaluate the value of juvenile arthritis MRI scoring as a sensitive measure regarding destructive changes, further international collaboration is warranted, especially among research centres with access to more severely affected JIA patients. Furthermore, collaboration should focus on developing an MRI atlas of healthy joints in children, obtaining agreement on an optimal imaging protocol for the knee and further validating scoring methods. Interaction between researchers and health professionals in JIA imaging is essential to obtain international consensus and continuous improvement of MRI outcome measures. Such collaboration is expected to be very fruitful under the umbrella of an international, well-accepted collaborative international group such as the Outcome Measures in Rheumatology working group.

According to the Quantification Imaging Biomarker Alliance of the Radiological Society of North America, quantitative imaging techniques covering the full spectrum of imaging have to be developed and validated to provide more objective tools to measure disease activity throughout all fields of medicine [38]. With respect to this, the Quantification Imaging Biomarker Alliance has formed the Contrast-enhanced Ultrasound Working Group to explore US contrast agent applications and use specific quantification software to evaluate disease activity and increase objectivity of the results of the US technique.

Forthcoming research is expected to shed more light on the suitability of advanced quantitative MRI techniques for evaluating inflammatory and destructive changes in the JIA knee, including dynamic contrast-enhanced MRI, T2-mapping and diffusion-weighted imaging [39–44]. Currently these advanced imaging techniques are used particularly in the context of research and to a lesser extent in daily practice. The exact value of advanced MRI techniques in children with JIA has to be determined in larger prospective studies. To be viable in daily practice, these imaging techniques should be sensitive to change on which evidence is limited [45]. It is important to further develop and implement advanced imaging techniques in clinical practice. For example, the contrast-free approach of diffusion-weighted imaging is highly desirable in clinical practice because it could substantially improve patient care by optimizing MRI feasibility in paediatric JIA patients; moreover, establishing normal values for the MRI atlas of healthy joints is less ethically compromising if contrast-free imaging is available.

## Conclusion

In this paper, we discussed the status of imaging the JIA knee. In the last decade a number of important steps have been made in the development of imaging outcome measures in children with JIA knee involvement. Ultrasound is undergoing a fast validation process, which should be accomplished within the next few years. The validation processes of MRI as an imaging biomarker for clinical trials in the JIA knee are at an advanced stage, with important data forthcoming from both single-centre as well as international multi-centre studies on the feasibility, reliability and validity of the Juvenile Arthritis MRI Scoring system. Moreover, data are emerging in both US and MRI on the normal appearance of the growing knee joint. However, future research is clearly needed, especially within the scope of evaluating the value of Juvenile Arthritis MRI Scoring as a sensitive measure for assessing destructive changes. The ultrasound score needs further validation, as well. Moreover, further validation is needed for promising advanced quantitative US and MRI techniques for the evaluation of inflammatory and destructive changes in JIA.
